# Analgesic Efficacy of Ultrasound-Guided Erector Spinae Plane Block in Patients with Extrahepatic Portal Venous Obstruction Undergoing Splenectomy: A Randomized Controlled Trial

**DOI:** 10.7759/cureus.81758

**Published:** 2025-04-05

**Authors:** Suruchi Ambasta, Prateek Bais, Chetna Shamshery, Ashish Kannaujia, Prabhaker Mishra, Keshav Garg, Swagat Mahapatra, Shivani Rastogi

**Affiliations:** 1 Anesthesiology, Sanjay Gandhi Postgraduate Institute of Medical Sciences, Lucknow, IND; 2 Biostatistics and Health Informatics, Sanjay Gandhi Postgraduate Institute of Medical Sciences, Lucknow, IND; 3 Anesthesiology, Healthworld Hopsital, Durgapur, IND; 4 Orthopedic Surgery, Dr. RML Institute of Medical Sciences, Lucknow, IND; 5 Anesthesiology, Dr. RML Institute of Medical Sciences, Lucknow, IND

**Keywords:** analgesia, epidural analgesia, erector spinae plane block, paravertebral block, splenectomy, thrombocytopenia

## Abstract

Background: Postoperative pain is quite prevalent in patients undergoing splenectomy and shunt surgery for extrahepatic portal venous obstruction (EHPVO) via midline laparotomy incision. Most of these patients present with thrombocytopenia in the preoperative period. The presence of thrombocytopenia excludes the placement of epidural catheter for postoperative analgesia, which is considered the gold standard for laparotomies. Systemic opioids remain the cornerstone of pain management in such cases, but they have their side effects. Better alternatives need to be explored to improve postoperative pain management and recovery. The erector spinae plane block (ESPB) has an excellent risk-benefit ratio and has been used for a wide range of cases, from acute postoperative pain to chronic pain conditions.

Methodology: This was a randomized controlled trial conducted on 84 patients who underwent splenectomy with lienorenal shunt surgery under general anesthesia. Patients in the study group were given ESPB before extubation, while the control group was managed on conventional analgesics. The primary objective was postoperative opioid requirement by intravenous patient-controlled analgesia (PCA) in both groups. Secondary objectives were static and dynamic Numerical Rating Scale (NRS) scores, hospital stay duration, time first to rescue analgesia, and incidences of adverse events.

Results: Patients in the ESPB group had less requirement of fentanyl in the postoperative period (median of 100 µg as compared to 880 µg in control group in first 24 hours). Static and dynamic pain scores were also less in the ESPB group at all time points (*P *< 0.001). Adverse events were higher in the control group compared to the ESPB group.

Conclusions: Ultrasound-guided ESPB provides superior analgesia and recovery with fewer side effects than conventional analgesics.

## Introduction

Extrahepatic portal venous obstruction (EHPVO) is a vascular disorder of the liver due to the obstruction of extrahepatic portal veins [1], leading to portal hypertension (PHT) (54%) and upper gastrointestinal bleeding in a majority of patients [2]. Local factors like trauma, tumor, or inflammation predispose adults with EHPVO in 19%-64% of cases. These patients present with an increased incidence of intense variceal bleeds, which are revenant and predisposed by fever or use of non-steroidal anti-inflammatory drugs (NSAIDs). Most idiopathic portal hypertension (IPH) cases are easily managed with endotherapies, thus avoiding surgery, but in EHPVO, surgery is the first line of management [3]. Midline laparotomy with splenectomy and proximal splenorenal shunt is the most commonly performed approach in these cases.

Patients undergoing abdominal surgical procedures present with severe pain following surgery, which may compromise breathing and lead to respiratory failure or pneumonia due to splinting and retained secretions. They are also prone to develop post-laparotomy pain syndrome, characterized by chronic abdominal pain beyond the expected recovery period [4]. In patients with EHPVO, platelet counts are generally low; thus, thoracic epidural analgesia is contraindicated in most cases. Traditional pain management with opiates and non-opioids like NSAIDs may have redundant systemic side effects [5], thus decreasing their popularity. A substantial dosage of opioids may cause respiratory depression, and inadequate coughing might lead to mechanical ventilation [6]. NSAIDs are inept to provide relief in severe pain and may cause gastrointestinal bleeding and are thus better avoided.

A felicitous analgesic alternative is the need of the hour, which can compensate for the shortcomings of the aforementioned options. Fascial plane blocks provide pain relief in a vast armamentarium of cases, ranging from acute pain in the perioperative period to chronic pain. These blocks are easy to perform with efficacious analgesia and lower complication rates. Forero et al. [7] described this technique almost a decade back in 2016 for treating chronic pain in thoracic neuropathies and acute postoperative pain in thoracic surgeries, but research in this area is mostly limited to case reports and case series [8]. Erector spinae plane block (ESPB) has emerged as a perfect substitute during contraindications to neuraxial or paravertebral blocks like patient refusal [9,10], low platelet counts [11,12], ongoing antiplatelet, [13] anticoagulant treatments [14], or coagulopathy [15]. ESPB is advantageous over conventional techniques performed close to the neuraxial plane. The ultrasound visualization is relatively simple and distant from surgical incision sites. Complications are less as pleura and major vascular structures are far away from the needle, thus increasing the safety margin and reducing complication rates [8]. ESPB has been proven effective in providing analgesia from cervical to lumbar levels. Though it may not be the primary choice, it is best suited as an alternative where the preferential methods pose risk or are contraindicated [8]. In the EHPVO patient group, epidural anesthesia, which is the gold standard, becomes controversial due to thrombocytopenia [11,12].

The erector spinae muscle (ESM) is a complex formed by three muscles, namely the spinalis, longissimus thoracis, and iliocostalis, running together in the back in a vertical orientation. After identifying the transverse process, a local anesthetic (LA) is deposited in the plane deep to the ESM. There is extensive craniocaudal drug spread, which diffuses anteriorly and laterally, covering the paravertebral, epidural, and intercostal spaces [16-18] and providing analgesic coverage from C7-T2 to L2-L3 [19]. The basis of this study is formed by the paucity of trials comparing ESP with the control group in open laparotomies, and as per our knowledge, no randomized trial has been conducted in splenectomy with shunt surgery. We performed a single-shot, bilateral ESPB using ropivacaine with dexmedetomidine as an adjuvant. Two groups were compared to evaluate the effects of ESPB in conjunction with conventional pain management protocols. This study aims to determine the opioid consumption postoperatively in the ESPB and control groups.

## Materials and methods

Patient population

**Figure 1:**
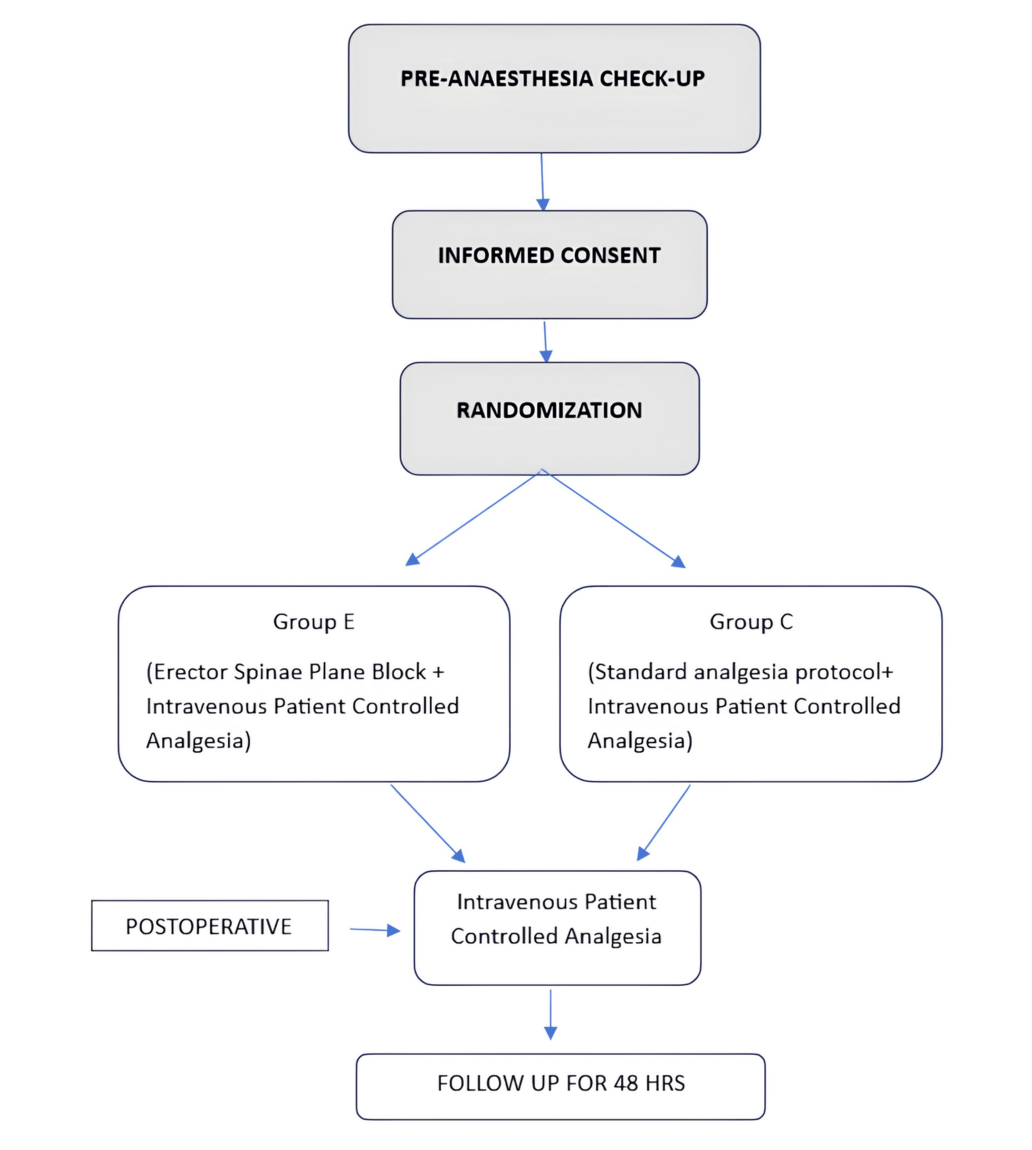
Study protocol flowchart.

**Figure 2:**
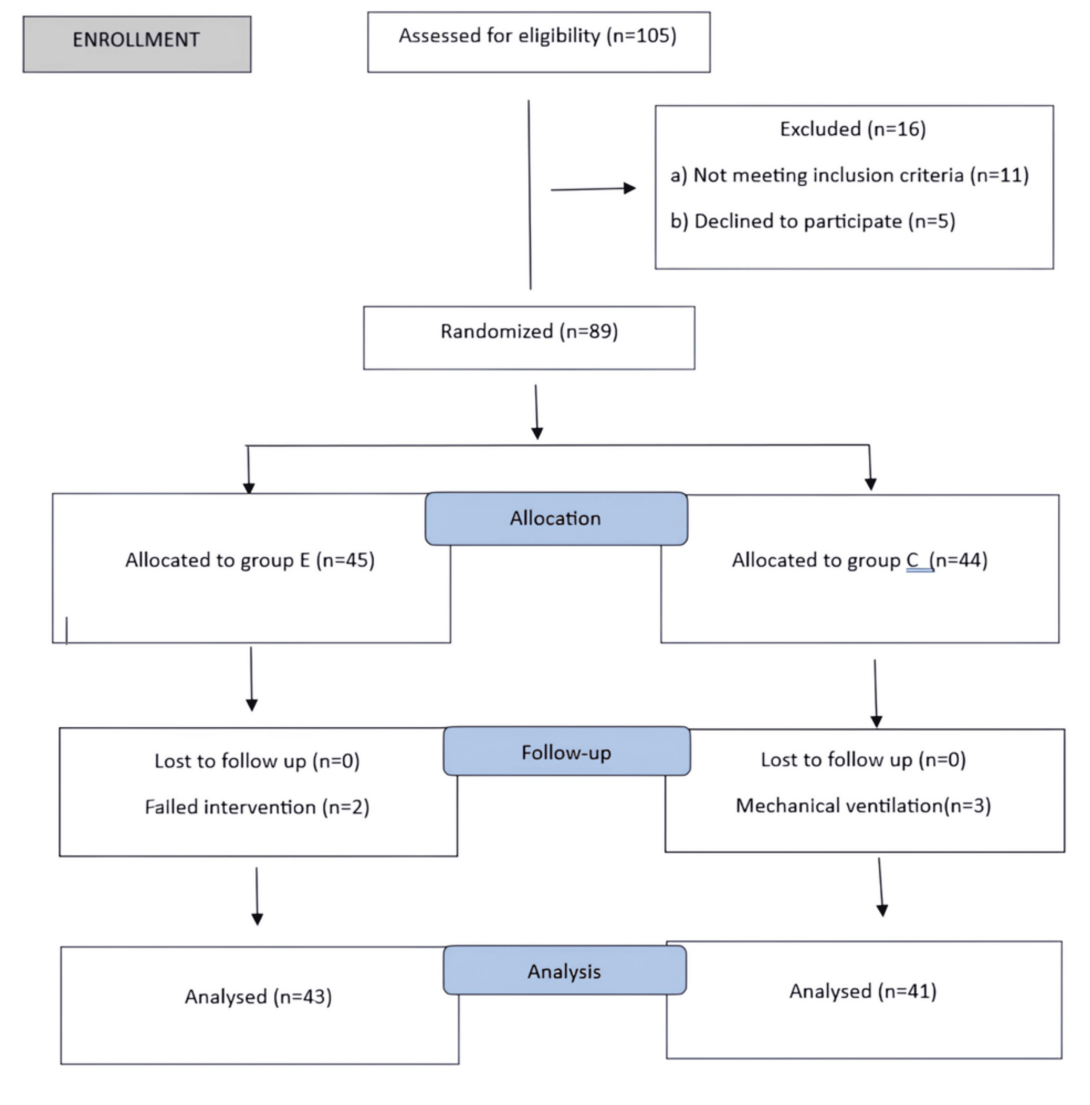
Consort flow diagram of the present study.

This was a prospective, randomized controlled trial (RCT) comparing patients undergoing splenectomy with proximal splenorenal shunt, with or without the addition of ESPB. After obtaining approval from the Institute Ethics Committee (2021-57-IP-119), we prospectively recruited patients undergoing elective splenectomy with lienorenal shunt at a tertiary care hospital in North India. The trial was registered prospectively in the Clinical Trials Registry of India (CTRI/2024/01/061935). The principles of the Declaration of Helsinki were followed. Patients over 18 years of age with an American Society of Anesthesiologists (ASA) physical status of I or II scheduled for elective open splenectomy with lienorenal shunt were included. Patients were excluded if they refused to participate, had a history of allergy to any medications, had an infection at the block site, had coagulopathies, or had a history of pre-existing chronic pain requiring medication. Written informed consent was obtained from all participants during the preoperative assessment. Patients were allocated to Group E or Group C using a computer-generated random number table, with 45 and 44 patients in each group, respectively (Figures 1, 2). Concealment was done using sequentially numbered, opaque, sealed envelopes. It was not possible to blind the anesthesiologist performing the block, but all participants and the anesthesiologist responsible for data collection were unaware of group allocation. Group E received ultrasound-guided bilateral ESPB in the lateral position at the T8 level. The block was administered after surgery but before extubation. Group C received general anesthesia along with conventional analgesia, including IV fentanyl and paracetamol. Both groups received IV PCA pumps in the postoperative period.

Anesthesia technique

All patients were educated on the Numeric Rating Scale (NRS) for pain (0 to 10, with 0 indicating no pain and 10 indicating the worst possible pain), and the use of the patient-controlled analgesia (PCA) device was demonstrated during the preoperative period. In the operating theater, standard monitors as per ASA guidelines were attached, and vitals were recorded. All patients received general anesthesia. Induction was achieved using intravenous (IV) fentanyl (2-3 μg/kg) and propofol (2 mg/kg). After confirming adequate ventilation with a bag-mask, muscle relaxation was achieved using IV atracurium (0.5 mg/kg), followed by tracheal intubation. Patients were placed on mechanical ventilation with a 50% air-oxygen mixture, and anesthesia was maintained with sevoflurane to achieve a minimum alveolar concentration (MAC) of 1.0 to 1.2. End-tidal carbon dioxide (EtCO2) was kept between 30 and 35 mmHg. IV paracetamol (1 g) was administered to all patients after induction by our institutional protocol. Intermittent fentanyl boluses of 1-2 μg/kg were given at regular intervals to maintain vitals near baseline values. All patients received injection ondansetron 0.1 mg/kg before extubation, and reversal of neuromuscular blockade was done using IV neostigmine (50 µg/kg) and glycopyrrolate (10 µg/kg). Group E patients received ESPB before extubation, while Group C patients were given conventional analgesia.

ESPB procedure

Patients received ultrasound-guided bilateral ESPB at the T8 level with 20 ml of 0.375% ropivacaine and dexmedetomidine 25 μg on each side, administered after surgery and before extubation. Patients were placed in the left lateral position for block placement. After painting and draping, a linear ultrasound transducer (Sonosite Edge II, 6-13 MHz) was placed in a parasagittal longitudinal orientation, 2-3 cm lateral to the midline. The tip of the transverse process of the T8 vertebra was identified beneath the trapezius and ESM. The rhomboid muscle was not present at this level, which aided in clear identification. An in-plane technique was used, with the needle advanced in a cranio-caudal direction, traversing the skin, subcutaneous tissue, trapezius, and erector spinae muscle to contact the transverse process. The needle was then slightly withdrawn, and its position was confirmed by injecting a small amount of saline. The drug was then administered, and its spread was visualized, separating the erector spinae muscle from the transverse process. A total of 20 mL of 0.375% ropivacaine (local anesthetic) with 25 μg of dexmedetomidine as an adjuvant was given on each side.

In the postoperative recovery unit, all patients, regardless of group allocation, were provided with an IV PCA pump (Smiths Medical 6300, Minneapolis, MN). The PCA contained fentanyl, delivered exclusively as demand doses. The infusion was prepared by diluting 10 mL of fentanyl injection (50 μg/mL) in 90 mL of 0.9% normal saline, resulting in a final concentration of 10 μg/mL of fentanyl. Each dose comprised 2 mL (i.e., 20 μg) of fentanyl. The lockout interval was set at 15 minutes, allowing a maximum of four doses (80 μg) per hour. Both groups of patients received IV paracetamol every six hours, as per our institutional protocol. Patients with static NRS > 4 or dynamic NRS > 5 were given Injection Diclofenac 1 mg/kg IV, diluted in 100 mL of 0.9% normal saline and administered slowly over 15 minutes, and were excluded from the study.

Variables and measurements

We collected demographic data, intraoperative and postoperative opioid consumption up to 48 hours, time to first rescue analgesia, static and dynamic NRS scores, and postoperative incidences of nausea, vomiting, and other adverse events such as hematoma, urinary retention, transfer to intensive care units for mechanical ventilation, or local anesthetic systemic toxicity (LAST). A few other parameters, such as surgical duration, resumption of peristalsis, and hospital stay, were also noted.

Statistical analysis

Data were presented as mean ± standard deviation (SD) along with the median. Data were considered normally distributed when the SD was less than half the mean value. To compare means between two independent groups, the independent samples t-test was used, whereas the chi-square (χ²) test was applied to compare proportions of categorical variables. Repeated measures analysis of variance (ANOVA), followed by multiple comparisons, were done using Bonferroni corrections. All statistical analyses were performed using SPSS software for Windows, version 23.0 (IBM Corp., Armonk, NY) and Stata 16 (StataCorp LLC, College Station, TX). A *P*-value < 0.05 was considered statistically significant.

Sample size

A minimum of 34 participants per group was required, assuming this would allow detection of a Cohen’s d effect size of 0.8 (considered a large effect size) for postoperative fentanyl requirements in the first 24 hours, with a power of 90% and an alpha error of 0.05. The sample size was calculated using G*Power software, version 3.1.9.7. In this study, the retrospective Cohen’s d effect size was found to be 2.3, with a power of 99.9%. We initially targeted a sample size of 45 patients per group, and after exclusions and dropouts, the final analysis was conducted on 84 patients.

## Results

A total of 105 patients were assessed for eligibility. Eleven were excluded, and five declined to participate. Eighty-nine patients were randomized into Group E (*n* = 45) and Group C (*n* = 44). The final analysis was performed on 84 patients due to failed intervention (ESPB) or the need for mechanical ventilation in the postoperative period (Figure 2).

Demographic data (age, gender, body mass index [BMI], and duration of procedure) are summarized in Table 1. Both groups had a similar demographic profile, and the surgical duration was also comparable between them. Group E showed earlier resumption of peristalsis and a significantly shorter hospital stay duration (*P* < 0.05) (Table 1).

**Table 1:** Comparison of demographics, surgical duration, resumption of peristalsis, and length of hospital stay. Data were presented as mean ± standard deviation (median) and compared using the independent samples t-test. A *P*-value < 0.05 was considered statistically significant. *Data were presented as numbers (%) and compared using the chi-square test. A *P*-value < 0.05 was considered statistically significant.

Variables	Group E (*n* = 43)	Group C (*n* = 41)	Test statistics	*P*-value
Age (years)	26.37 ± 9.35 (25)	27.61 ± 8.64 (25)	0.630	0.335
Male sex: Number (%)*	28 (65.1)	25 (60.9)	4.198	0.344
Weight (kg)	49.63 ± 8.44 (49)	48.29 ± 7.03 (47)	0.789	0.376
Height (cm)	156.35 ± 8.29 (156)	156.54 ± 7.16 (155)	0.111	0.932
BMI (kg/m^2^)	20.20 ± 2.54 (20)	19.66 ± 2.15 (19.57)	1.053	0.298
Surgical duration (minutes)	351.86 ± 46.2 (340)	341.46 ± 45.31 (320)	1.041	0.293
Resumption of peristalsis	9.7 ± 1.67 (9)	12.8 ± 1.69 (13)	8.469	<0.001
Length of hospital stay (days)	8.14 ± 1.68 (8)	10.71 ± 2.72 (10)	5.169	<0.001

**Figure 3:**
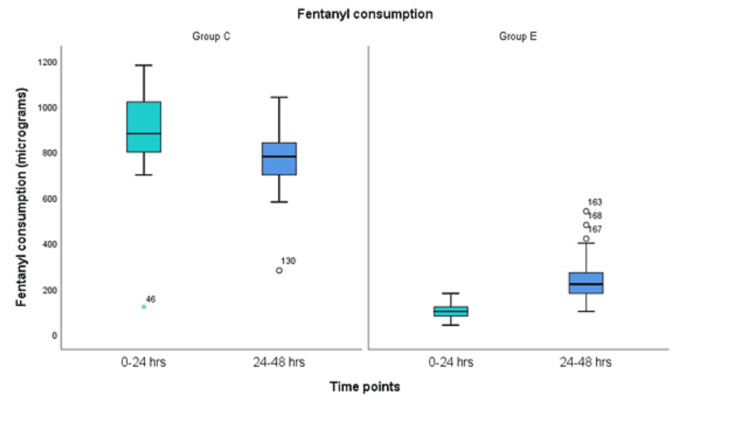
Comparison of fentanyl consumption in the postoperative period.

**Figure 4:**
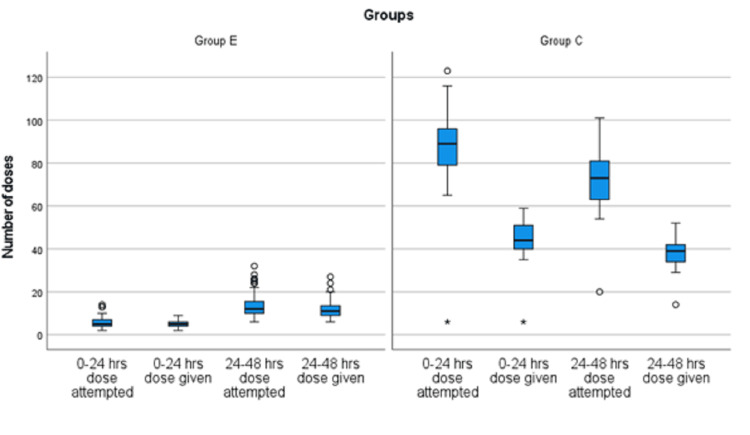
Comparison of dose attempted and dose given in the postoperative period.

Table 2 demonstrates opioid consumption during the intraoperative and postoperative periods. Both groups were comparable in terms of intraoperative opioid usage. However, the median 24-hour fentanyl consumption was 100 µg in Group E and 880 µg in Group C, which was statistically significant (*P* < 0.001), reflecting an almost eightfold higher requirement in Group C (Table 2, Figure 3). The number of attempts for fentanyl delivery via IV PCA was also recorded in both groups, with Group C showing a significantly higher number of attempts compared to Group E during the first 24 hours (Figure 4). During the 24- to 48-hour period, the median fentanyl consumption was 380 µg in Group E and 780 µg in Group C, which was statistically significant (*P* < 0.001). This indicates that the ESPB group required less than half the dose compared to the control group (Table 2, Figure 3). The number of IV PCA attempts was more than three times higher in the control group (Figure 4). The median time to first rescue analgesia with fentanyl was six hours (IQR: 6-8) in Group E and one hour (IQR: 1-2) in Group C.

**Table 2:** Details of opioid requirements in study participants. Data were presented as mean ± standard deviation (median) and compared using the independent samples t-test. A *P*-value < 0.05 was considered statistically significant. Independent samples t-test (t-value) and chi-square test (χ² value) were used to calculate test statistics and corresponding significance levels. *Values were presented as median (interquartile range) and analyzed using the Mann-Whitney U test.

Variables	Group E (*n *= 43)	Group C (*n* = 41)	Test statistics	*P*-value
Intraoperative opioid	237 ± 59.47 (221)	249.27 ± 40.68 (260)	14.942	0.283
Time to first rescue analgesia (hours)*	6 (6-8)	1 (1-2)	14.15	<0.001
0-24 hours postoperative period				
Dose attempted	5.88 ± 2.77 (5)	87.51 ± 18.8 (89)	27.526	<0.001
Dose given	5.14 ± 1.82 (5)	44.22 ± 9.02 (44)	27.216	<0.001
Total fentanyl used (μg)	102.79 ± 36.41 (100)	884.39 ± 180.42 (880)	27.216	<0.001
24-48 hours postoperative period				
Dose attempted	28.37 ± 12.89 (24)	71.76 ± 14.12 (73)	23.847	<0.001
Dose given	21.0 ± 8.07 (19)	37.95 ± 6.39 (39)	20.773	<0.001
Total fentanyl used (μg)	421.6 ± 161.4 (380)	761.46 ± 127.15 (780)	20.764	<0.001

**Figure 5:**
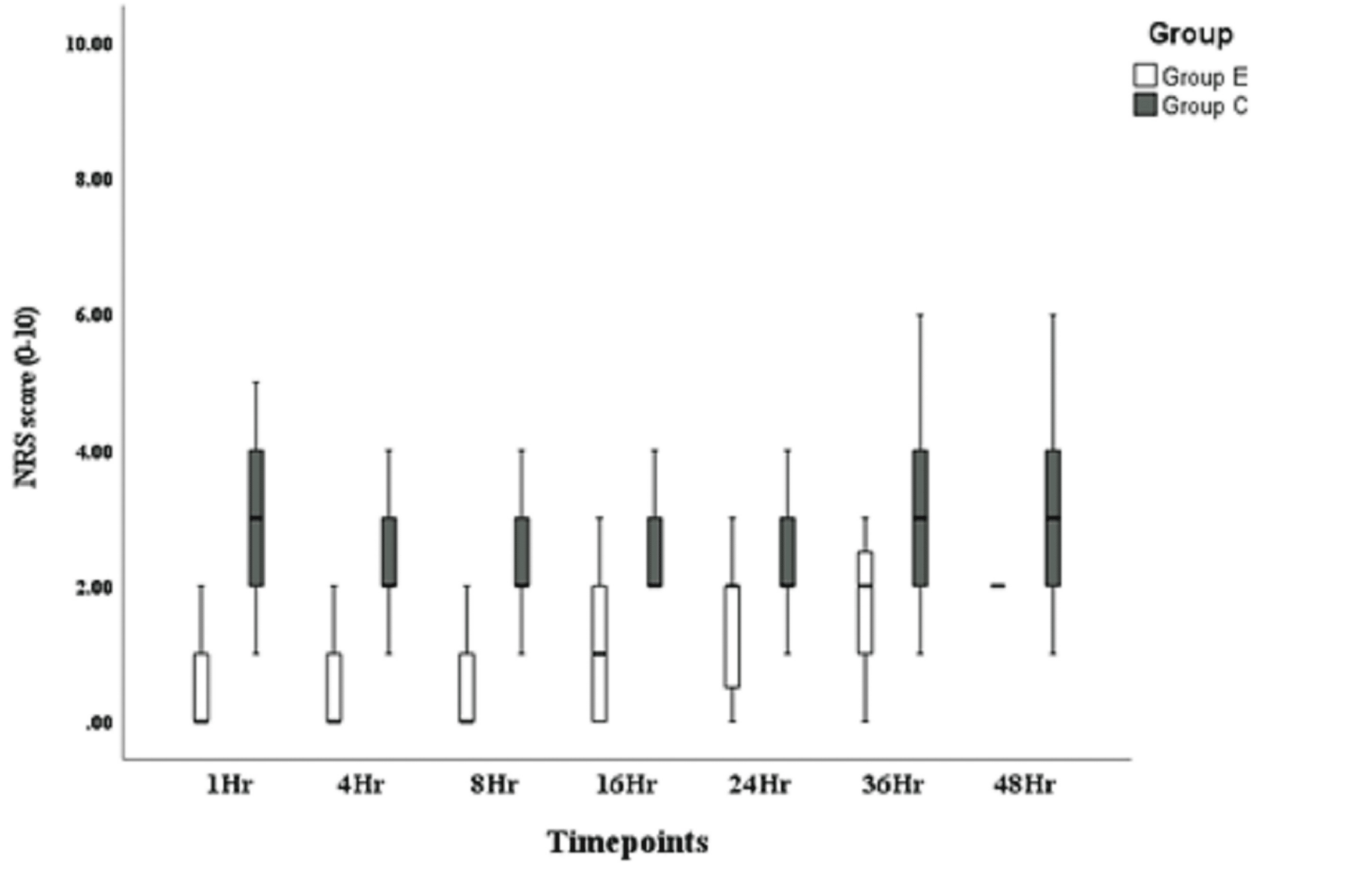
Numeric Rating Scale (NRS) at rest.

**Figure 6:**
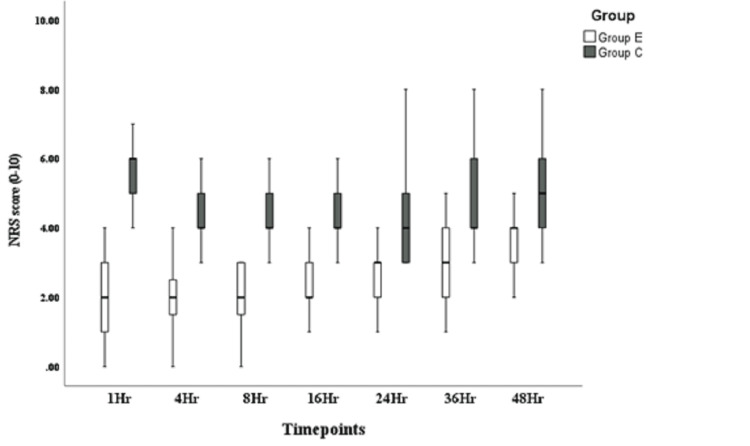
Numeric Rating Scale (NRS) during movement.

Table 3 presents a comparison of static and dynamic NRS scores assessing pain in both groups at various time points (1, 4, 8, 16, 24, 36, and 48 hours) (Figures 5, 6). There was a significant difference at all time points, with the study group recording lower pain scores in both static and dynamic values.

**Table 3:** NRS scores comparison between two groups. Data were presented as mean ± standard deviation (median) and compared using the independent samples t-test ($). Two-way repeated measures ANOVA (##) and repeated measures ANOVA (#) were used for longitudinal comparisons, with multiple comparisons adjusted using Bonferroni correction. Independent samples t-test (t value) and chi-square test (χ² value) were used to calculate test statistics and significance levels. A *P*-value < 0.05 was considered statistically significant. ANOVA, analysis of variance; NRS, Numerical Rating Scale

Variables	Group S (*n *= 43)	Group C (*n *= 41)	Test statistics	*P*-value
NRS (Static)				
1 hour	0.6 ± 0.76 (0)	3.22 ± 1.06 (3)	13.031	<0.001
4 hours	0.51 ± 0.7 (0)	2.46 ± 0.92 (2)	10.854	<0.001
8 hours	0.58 ± 0.73 (0)	2.39 ± 1.12 (2)	8.830	<0.001
16 hours	0.93 ± 0.91 (1)	2.78 ± 0.96 (2)	9.046	<0.001
24 hours	1.28 ± 0.88 (2)	2.68 ± 1.25 (2)	5.910	<0.001
36 hours	1.77 ± 0.95 (2)	2.76 ± 1.18 (3)	4.226	<0.001
48 hours	1.98 ± 0.71 (2)	3.1 ± 1.09 (3)	5.614	<0.001
*P*-value^#^	<0.001	0.002		<0.001^##^
*F*-value	23.86	5.49		
Multiple comparisons (*P *< 0.05)	1 hour-4 hours, 8 hours	1 hour-24 hours, 36 hours, 48 hours		
NRS (Dynamic)				
1 hour	1.77 ± 1.11 (2)	5.54 ± 1.4 (6)	13.646	<0.001
4 hours	1.98 ± 0.86 (2)	4.12 ± 1 (4)	10.495	<0.001
8 hours	1.98 ± 0.83 (2)	4.49 ± 1.21 (4)	11.514	<0.001
16 hours	2.33 ± 1.11 (2)	4.63 ± 1.07 (4)	9.735	<0.001
24 hours	2.7 ± 0.99 (3)	4.51 ± 1.5 (4)	6.569	<0.001
36 hours	3.14 ± 1.04 (3)	4.66 ± 1.37 (4)	5.743	<0.001
48 hours	3.7 ± 0.96 (4)	5.05 ± 1.32 (5)	5.330	<0.001
*P*-value	<0.001^$^	<0.001^$^		<0.001^##^
*F*-value	23.99	7.60		
Multiple comparisons (*P *< 0.05)	1 hour-24 hours, 36 hours, 48 hours	1 hour-4 hours, 8 hours, 12 hours, 16 hours, 24 hours		

Table 4 shows the incidence of adverse events with eight (18.5%) patients in Group E and 20 (48.7%) patients in Group C with complaints of postoperative nausea and vomiting. There were two (4.65%) cases of urinary retention in Group E and four (9.76%) cases in Group C. Other complications like hematoma formation or LAST were not reported in either group.

**Table 4:** Incidence of adverse effects in study groups. Data were presented as numbers (%), and comparisons were made using Fisher’s exact test. As Fisher’s exact test did not yield a test statistic, the corresponding chi-square values were reported. A *P*-value < 0.05 was considered statistically significant. LAST, local anesthetic systemic toxicity

Adverse events		Group E (*n* = 43)	Group C (*n *= 41)	Test statistics	*P*-values
Nausea and vomiting		8 (18.6%)	20 (48.7%)	21.143	<0.001
Urinary retention		2 (4.65%)	4 (9.76%)	0.825	0.364
Hematoma		0 (0%)	0 (0%)		0.99
LAST		0 (0%)	0 (0%)		0.99

## Discussion

We performed a randomized controlled study in patients undergoing splenectomy with shunt surgery. The intervention done was ultrasound-guided bilateral ESPB for postoperative analgesia. Patients in both groups were comparable in demographic parameters and surgical duration. There was a significant reduction in opioid consumption and lower NRS scores in the ESPB group in the postoperative period up to 48 hours.

Regional anesthesia plays a pivotal role in abdominal surgery as an inevitable component of multimodal analgesia [20] and changes outcomes in patient management and recovery [21]. However, the choice varies and remains highly debatable regarding safety, efficacy, appropriateness, and consistency. Though ESPB is quite popular in thoracic surgeries [22], its successful use as a postoperative analgesia option in open abdominal procedures has been published mostly as case reports and case series [23]. Bilateral ESPB has been used for many upper and lower abdominal surgical procedures, but very few RCTs have been published. Though thoracic epidural analgesia is still considered the gold standard or primary choice in open abdominal surgeries for postoperative analgesia [24], its role remains controversial in EHPVO patients as they present with low platelet count in the preoperative period. Complications like epidural hematoma have led to the decreased popularity of epidural analgesia in these patients [25,26]. Also, studies have proven that epidural analgesia does not show any benefit in terms of hospital stay duration or postoperative complications like ileus after open abdominal surgery [27].

The thoracic paravertebral block is a frequent choice in postoperative analgesia in breast and thoracic surgery, but its role in abdominal surgery is not yet established due to limited evidence [28]. Another important aspect is the expertise required as it is an advanced regional anesthetic technique consuming more time and with a high incidence of pneumothorax [29]. Intravenous opioids have been traditionally used in all these cases, which have numerous undesirable systemic side effects [30-32]. Thoracic ESPB has lower complication rates and a lesser learning curve as compared to thoracic paravertebral block or thoracic epidural analgesia [33]

Nagaraja et al. [34] studied 50 patients undergoing cardiac surgery to compare the continuous thoracic epidural with bilateral ESPB and demonstrated consistently lower NRS scores in the ESPB group. An RCT by Xu et al. [35] concluded that ESPB provided the same degree of analgesia for static pain compared to thoracic paravertebral block for laparoscopic nephrectomy. Puncture time (42.1 s vs. 56.8 s) was shorter, and the first attempt puncture success rate was higher (83% vs. 58%) with lower pain during block procedure (2 vs. 3) in the ESPB group [35]. When compared to other abdominal wall blocks like the transversus abdominis plane (TAP), rectus sheath, and quadratus lumborum blocks, ESPB provides extensive craniocaudad coverage with a low risk-benefit ratio and no intervention near the surgical field in thoracoabdominal surgeries [36]. ESPB reliably covers the upper, lower, as well as the lateral abdominal walls with a single injection, which is potentially advantageous as compared to other blocks. It provides both visceral and somatic analgesia [37] and is convenient in the postoperative period, irrespective of surgical dressings or tissue disruption during surgery [10]. ESPB has the potential to create a paradigm shift from the conventional analgesics and traditional regional anesthesia techniques in abdominal surgeries. During the last decade, ESPB has gained momentum due to its safe and simple approach.

We performed ultrasound-guided bilateral ESPB just before extubation and observed a significant reduction in opioids consumed in the postoperative period. Patients in the study group also had lower NRS scores at all time points with lesser incidence of adverse events like postoperative nausea and vomiting (PONV). These findings are consistent with previous research by Dubilet et al. [38], who studied the effect of ESPB in open oncologic abdominal surgery. Meta-analysis by Fu et al. [39] in lumbar spine surgery concluded that ESPB decreases the opioid consumption, rescue analgesic requirement, and number of attempted doses by PCA pump, which is in concordance with our study. Our patients in the ESPB group were also discharged early (median 8 days vs. 10 days in the control group). Gürkan et al. [40] studied the effect of ESP block in 50 breast surgery patients and concluded decreased morphine consumption by 65% at 24 hours compared to the control group (5.76 ± 3.80 mg vs. 16.60 ± 6.92 mg). Patients in the ESPB group had 80% lower fentanyl consumption, with a median 24-hour usage of 100 μg compared to 880 μg in the control group.

In ESPB, both somatic and visceral pain is decreased due to the local anesthetic spread in the paravertebral space and later to the epidural space through the intervertebral foramina [18,19,41]. The transverse process is selected as per the surgical requirements, which should block the posterior branch of the corresponding spinal nerve [39]. A cadaveric study demonstrated the extensive spread from C7-T2 transverse processes to L2-L3 transverse processes with injection of 20 mL of fluid at T7 vertebral level [19]. We performed the block at T8 with 20 mL of local anesthetic, and though we did not assess the dermatomal spread, the decreased NRS Scores after midline laparotomies in our patients proved the somatic and visceral coverage similar to the aforementioned studies.

We used dexmedetomidine as an adjuvant in our study. It is a highly selective α2 adrenergic receptor agonist with analgesic, sedative, and anxiolytic effects and no respiratory depression [42]. Recent studies have shown that it causes local vasoconstriction and prolongs the block duration when used as an adjunct to local anesthetics [43]. Delayed absorption of local anesthetics leads to prolonged effect [44].

Perineural dexmedetomidine (100 μg), when added to ropivacaine, doubled the duration of an ulnar nerve block compared to ropivacaine alone [45]. Analgesic duration is prolonged, with an overall decrease in opioid consumption, with the addition of 0.5 μg/kg dexmedetomidine to ropivacaine [46]. In a meta-analysis of 32 trials by Vorobeichik et al. [43], it was found that dexmedetomidine extends both sensory and motor block durations and decreases the onset time with a dose of 50-60 μg without any cardiovascular effects. A recent randomized controlled study on posterior lumbar spine surgery demonstrated a prolonged duration of sensory block [47]. Our study used a total dose of 50 μg of dexmedetomidine, with 25 μg administered on each side, and the prolonged effect observed with single-shot ESPB was comparable to that reported in the aforementioned studies. Although 100 μg of dexmedetomidine can be safely used perineurally [43], we opted for a dose of 50 μg (25 μg on each side). The effect of the block was conveniently increased to 24-36 hours, with an increased demand for rescue analgesia observed after 36 hours. In a recent study by Dubilet et al. [38], the analgesic effect following a single-shot ESPB lasted up to 12 hours, after which opioid consumption increased in the study group, and VAS scores were elevated. This proves the efficacy of dexmedetomidine in prolonging block duration in our study, which extended to almost 36 hours. Yi-Han et al. [47] observed that the addition of dexmedetomidine to ropivacaine for ESPB following posterior lumbar spine surgery increased the sensory block duration and provided prolonged analgesia. Gao et al. [48] proved that the addition of dexmedetomidine (1 µg/kg) to 0.5% ropivacaine increased the block duration by almost 120% in breast surgeries. A recent study in video-assisted thoracoscopic lobectomies revealed that ESPB with 1 μg/kg of dexmedetomidine and 0.33% ropivacaine provided superior postoperative analgesia [49]. The findings of our study were consistent with the aforementioned research regarding dexmedetomidine use and the prolonged duration of ESPB.

Our study showed similar NRS scores and postoperative opioid consumption to those reported by Ueshima et al. [50]. They conducted a retrospective study on lumbar spine surgery patients and observed lower NRS scores and reduced fentanyl consumption in the ESPB group compared to the control group. In a meta-analysis by Fu et al. [39], ESPB significantly reduced the static and dynamic pain scores within 48 hours after lumbar spinal surgery when compared to the control group. Our patients demonstrated a similar profile, with significantly lower NRS values at all time points and a considerable decrease in postoperative opioid consumption. Prospective RCTs by Kot et al. [8] comparing ESPB with a control group revealed reduced pain scores and decreased need for supplemental analgesia, similar to our study.

It is difficult to determine the failure of interfascial blocks as evaluation has multiple components, and assessment of dermatomal spread alone is not sufficient to comment on block performance [51]. We could not assess the dermatomal spread, and our block success was determined by NRS scores in the postoperative period. Our study had two cases of block failure, and they were excluded from the analysis.

Only a few RCTs performed in lumbar spine surgery patients have studied the number of PCA attempts and showed the lesser number of attempts in the ESPB group as compared to the control group (P=0.004) [39]. This is similar to our study results where ESPB patients pressed PCA pumps much less frequently than the control group (median 5 as compared to 89) in the first 24 hours. In 24-48 hours, the number of attempts increased in the ESPB group but still was significantly less than the control group. (median of 24 vs 73). We recorded increased opioid consumption after 36 hours, even in the ESPB group, and that can be described as the wearing off the block effect. NRS pain scores and opioid consumption increased after 36 hours, but the median opioid consumption was still one-third in the ESPB group.

Study group patients had considerably lower PONV incidence, which is in concordance with a previous study by Dubilet et al. [38]. Postoperative complications related to the ESPB were not seen in our study, indicating that it is a safe and easy option for postoperative pain management in splenectomy and shunt patients with midline laparotomy [38]. Another RCT by Loganathan et al. [52] performed in living donor nephrectomy did not show a statistically significant difference in both the groups. In their study on laparoscopic nephrectomy, Loganathan et al. administered a bilateral block and reported no complications during its administration or in the postoperative period, similar to our study.

We educated our patients about postoperative pain and the use of IV PCA, which led to their pain-free recovery. None of our patients in the ESPB group complained of rebound pain even after wearing the block effect, as described in previous studies [38].

Limitations

Our study has a few limitations. We were unable to blind the anesthesiologist performing the block; however, data were meticulously recorded by another anesthesiologist who was blinded to group allocation. Additionally, we evaluated only a single dose of dexmedetomidine as an adjuvant. Further studies are needed to determine the optimal dose of adjuvants for ESPB. We could not study the extent of dermatomal sensory block following the procedure. Correlation between gender and postoperative analgesic requirements was not checked. Patient satisfaction was not assessed in any of the groups, which could have added value to the study. In addition, our study did not assess the preoperative anxiety and depression status of patients, which could introduce bias by influencing pain perception scores. We hope that future studies will address these factors.

## Conclusions

Ultrasound-guided ESPB is an effective and easy-to-perform technique in midline laparotomies and can be used as an option in multimodal analgesia to reduce opioid consumption. It provides optimum analgesia with lower opioid consumption and decreased postoperative adverse events. The addition of an adjunct like dexmedetomidine increases the duration even after a single-shot block. We conclude that ESPB has been identified as a safe and effective approach, encouraging further studies in this direction to better guide clinical professionals.
